# Association between sarcopenia and osteoarthritis among the US adults: a cross-sectional study

**DOI:** 10.1038/s41598-023-50528-z

**Published:** 2024-01-02

**Authors:** Peng Peng, Jiawei Wu, Weihua Fang, Jiaqing Tian, Mincong He, Fangjun Xiao, Kun Lin, Xuemeng Xu, Wei He, Wengang Liu, Qiushi Wei

**Affiliations:** 1 Guangzhou University of Chinese Medicine, Guangzhou, People’s Republic of China; 2 Guangdong Research Institute for Orthopedics and Traumatology of Chinese Medicine, No. 261, Longxi Road, Liwan District, Guangzhou, 510378 People’s Republic of China; 3 Department of Orthopaedics, The Third Affiliated Hospital, Guangzhou University of Chinese Medicine, Guangzhou, People’s Republic of China; 4 Guangdong Provincial Second Hospital of Traditional Chinese Medicine, No. 60, Hengfu Road, Yuexiu District, Guangzhou, 510405 People’s Republic of China

**Keywords:** Medical research, Epidemiology

## Abstract

The association between sarcopenia and OA still presents many uncertainties. We aimed to assess whether sarcopenia is associated with occurrence of OA in US adults. We conducted a cross-sectional study consisting of 11,456 participants from National Health and Nutrition Examination Survey 1999–2006. Sarcopenia was defined by a low muscle mass. The skeletal muscle index (SMI) was calculated as the appendicular skeletal muscle mass divided by body mass indexes (BMI) or body weight. OA status was assessed by using self-reported questionnaire. We evaluated the association between sarcopenia and OA using multivariate regression models. In addition, subgroup and interaction analysis were performed. Sarcopenia was associated with OA when it was defined by the BMI-adjusted SMI (OR = 1.23 [95% CI, 1.01, 1.51]; *P* = 0.038) and defined by the weight-adjusted SMI (OR = 1.30 [95% CI, 1.10, 1.55]; *P* = 0.003). Subgroup and interaction analysis found that the strongest positive association mainly exists in smoker (OR = 1.54 [95% CI, 1.21, 1.95], Pint = 0.006), and this association is not significant in other groups. In conclusion, we found that sarcopenia was associated with occurrence of OA. Subgroup analysis revealed that the association between sarcopenia and OA was more pronounced in smoker. Further well-designed prospective cohort studies are needed to assess our results.

## Introduction

Sarcopenia is a degenerative and generalized skeletal muscle disease, which characterized by age-related decline of skeletal muscle combined with low muscle strength and performance^1^. Sarcopenia is known to be associated with negative outcomes, such as fragility fractures, frailty, disability and high mortality^2–4^. It has gradually become a global problem that brings a huge impact on economy and health.

In the past, the underlying mechanisms of sarcopenia has mainly focused on malnutrition, obesity, unnormal insulin signal, and Vitamin D deficiency^5–7^. Recently, low-grade local and systemic inflammation has been reported as leading to the development of sarcopenia. It is well known that inflammation is a key risk factors of osteoarthritis (OA). Meanwhile, sarcopenia and OA share a set of risk factors, such as aging, obesity, and diabetes^8,9^. In this regard, there is ongoing research involve in detecting of the association between sarcopenia and OA. A study from Korean suggested that low skeletal muscle mass was independently associated with knee OA^10^. However, the sample size was relatively small, and there is limited data on the association between sarcopenia and OA among the representative and large sample of US population. In addition, several studies have detected no association of sarcopenia with OA. A large longitudinal cohort study revealed that sarcopenia was not significantly associated with knee OA risk in both men and women^11^. However, this study involved primarily Caucasians, and its findings may not apply to other racial groups.

Taken together, the relationship between sarcopenia and OA still presents many uncertainties. More studies with representative and large samples are needed to better validate the association between sarcopenia and risk of OA. This study sought to explore the association between sarcopenia and OA in US adults using data from the National Health and Nutrition Examination Survey (NHANES) database.

## Methods

### Study population

**Figure 1:**
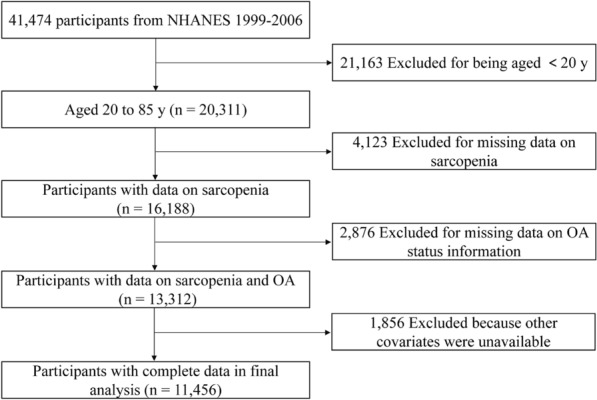
Flowchart of sample selection.

The NHANES database is an ongoing population-based national survey focusing on health and health-related behaviours of the US population. The NHANES database is available publicly at www.cdc.gov/nchs/nhanes. We collected data from 4 NHANES cycles, corresponding to a 8-year cross-section ranging from 1999 to 2006. In total, 20,311 participants aged over 20 years were included. We excluded participants without valid data for sarcopenia (no reliable dual-energy X-ray absorptiometry (DXA) and BMI data). After further excluding individuals with missing value for arthritis status information (n = 2,876) and other covariates (n = 1,856), 11,456 participants were enrolled for analysis (Fig. 1).

### Definition of sarcopenia

Skeletal muscle mass was determined for each participant using DXA whole-body scans (QDR-4500 Hologic scanner, Bedford, MA). Appendicular skeletal muscle mass (ASM (kg)) was defined as the sum of four limbs’ muscle mass. The skeletal muscle index (SMI) was calculated as the appendicular skeletal muscle mass divided by body mass indexes (BMI) or body weight (Wt). The cutoff values for low muscle mass were defined by the BMI-adjusted SMI was < 0.789 for males and < 0.512 for females, respectively^12^. In addition, we defined sarcopenia as an ASM/Wt less than one standard deviation (SD) below the sex-specific mean of young reference group^13^. The cut-off values of for low muscle mass were defined by Wt-adjusted SMI was 29.76% (ASM/Wt) for males and 22.31% (ASM/Wt) for females, respectively.

### Definition of OA status

OA status was assessed by a questionnaire survey^14^. Participants were asked: “Has a doctor or other health professional ever told you that you have arthritis?” Those who answered “no” were defined as without OA. If the answer is “yes”, the patients will be further to answer a follow-up question, “Which type of arthritis was it?” Those who self-reported “osteoarthritis” was defined as with OA.

### Covariates

Other covariates that could confound the association between sarcopenia and OA were included. Socio-demographic covariates included age, race/ethnicity (including non-Hispanic white, non-Hispanic black, Mexicane American, Other Hispanic, and other ethnicity), and education (including lower than high school, high school, and more than high school). Physical activity was assessed using the Physical Activity Questionnaire (PAQ) that is embedded in the NHANES survey, and was categorized as sedentary, low, moderate, and high^15^. Smoking status was determined based on a questionnaire, in which the patient answers the question “Have you smoked at least 100 cigarettes in your entire life?” Those who answered “yes” were defined as smoker. The other covariates included total protein, total cholesterol, HDL cholesterol, triglycerides, hemoglobin, serum calcium, and calf circumference. All examinations were carried out by well-trained medical experts. Information on each variable and acquisition process are publicly available at www.cdc.gov/nchs/nhanes.

### Statistical analysis

Continuous variables were presented as mean ± standard deviation and categorical variables as percentages. We grouped participants on the basis of their sarcopenia status and self-reported OA status. We further stratified low muscle mass based on either the BMI-adjusted SMI or weight-adjusted SMI. We used the ANOVA tests for continuous variables with a normal distribution and Kruskal–Wallis test for continuous variables without a normal distribution. The chi-square tests were used for categorical variables to assess the characteristics of the participants. Multivariate logistic regression analyses were performed to evaluate the relationship between sarcopenia with OA with odds ratio (OR) and corresponding 95% confidence interval (CI). We contrasted three models as follows: model 1, no adjustment for covariates; model 2, adjusted for age, gender, and race/ethnicity; model 3, additionally adjusted for education, physical activity, total protein, total cholesterol, HDL cholesterol, triglycerides, hemoglobin, serum calcium, calf circumference, and smoking status.

In addition, subgroup analyses were also conducted stratified by different age and gender. A two-sided *P* value < 0.05 was considered statistically significant. Statistical analyses were performed using the EmpowerStats (http://www.empowerstats.com, X&Y Solutions, Inc., Boston, MA) and statistical software packages R (http://www.R-project.org, The R Foundation).

### Ethics approval and consent to participate

The studies involving human participants were reviewed and approved by board of the National Center for Health Statistics. All the participants provided their written informed consent to participate in this study.

## Results

### Characteristics of participants

Table 1 summarized participant characteristics in subjects with non-sarcopenia and sarcopenia. According to the BMI-adjusted SMI, 1509 (13.17%) participants were diagnosed with sarcopenia. Participants with sarcopenia tend to be older, female, to have higher sedentary physical activity, and demonstrate differences in total cholesterol, triglycerides, HDL cholesterol, hemoglobin, and calf Circumference. In contrast, according to the weight-adjusted SMI, 3584 (31.28%) participants had sarcopenia. Participants with sarcopenia tend to be older, to have higher sedentary physical activity, and demonstrate differences in total cholesterol, triglycerides, hemoglobin, total protein, HDL cholesterol, serum albumin, and calf circumference.

**Table 1:** Baseline characteristics of the study subjects according to sarcopenia status.

	Sarcopenia defined SMI by ASM/BMI			Sarcopenia defined SMI by ASM/Wt		
	No sarcopenia (n = 9947)	Sarcopenia (n = 1509)	*P* value	No sarcopenia (n = 7872)	Sarcopenia (n = 3584)	*P* value
Age (years)	44.81 ± 16.81	59.43 ± 17.68	< 0.001	42.32 ± 15.94	56.43 ± 17.28	< 0.001
Gender (%)			0.003			0.246
Male	50.88	46.79		49.97	51.14	
Female	49.12	53.21		50.03	48.86	
Race (%)			< 0.001			< 0.001
Non-Hispanic White	49.54	43.54		45.39	56.14	
Non-Hispanic Black	21.63	2.72		25.14	5.97	
Mexican American	20.89	44.27		20.76	31.03	
Other Hispanic	4.20	5.63		4.69	3.74	
Other ethnicity	3.73	3.84		4.03	3.12	
Education (%)			< 0.001			< 0.001
Lower than high school	25.99	48.21		26.43	34.36	
High school	23.38	21.91		22.72	24.22	
More than high school	50.63	29.88		50.85	41.42	
Physical activity (%)			< 0.001			< 0.001
Sedentary	20.56	35.12		20.43	26.98	
Low	27.76	30.09		27.18	29.99	
Moderate	19.03	15.24		18.66	18.25	
High	32.65	19.55		33.73	24.78	
Total protein (g/dL)	7.36 ± 0.48	7.33 ± 0.49	0.101	7.38 ± 0.48	7.29 ± 0.48	< 0.001
Total cholesterol (mg/dl)	200.22 ± 42.08	208.24 ± 43.77	< 0.001	198.25 ± 42.15	207.92 ± 42.16	< 0.001
HDL cholesterol (mg/dl)	52.34 ± 15.61	51.66 ± 15.58	0.058	53.11 ± 15.72	50.43 ± 15.21	< 0.001
Triglycerides (mg/dl)	139.33 ± 121.69	168.93 ± 162.92	< 0.001	131.95 ± 117.03	168.01 ± 147.02	< 0.001
Hemoglobin (g/dL)	14.43 ± 1.50	14.33 ± 1.51	0.004	14.38 ± 1.51	14.49 ± 1.48	0.002
Serum albumin (g/dL)	4.32 ± 0.33	4.21 ± 0.33	< 0.001	4.35 ± 0.33	4.23 ± 0.32	< 0.001
Calf circumference (cm)	38.35 ± 4.00	37.22 ± 4.33	< 0.001	37.91 ± 3.85	38.84 ± 4.44	< 0.001
Smoking status (%)						< 0.001
Non-smoker	52.22	54.21		53.76	49.67	
Smoker	47.78	45.79		46.24	50.33	

Mean ± SD for continuous variables and *P* value was calculated by Kruskal–Wallis test. % for Categorical variables and *P* value was calculated by weighted chi-square test.

*SMI* skeletal muscle index, *ASM* appendicular skeletal muscle mass, *BMI* body mass index.

The characteristics of participants stratified by OA status are presented in Table 2. Among the 11,456 participants, 1055 were diagnosed with OA. Compared with the non-OA group, the OA group tend to be older and female. Participants with OA or non-OA were similar in calf circumference, while race/ethnicity, education, physical activity, total protein, total cholesterol, HDL cholesterol, triglycerides, hemoglobin, serum calcium, and smoking status were significantly different between these two groups.

**Table 2:** Baseline characteristics of the study subjects according to OA status.

	No OA (n = 10,401)	OA (n = 1055)	*P* value
Age (years)	44.94 ± 16.99	64.45 ± 13.63	< 0.001
Gender (%)			< 0.001
Male	51.96	34.41	
Female	48.04	65.59	
Race (%)			< 0.001
Non-Hispanic White	46.37	72.23	
Non-Hispanic Black	19.86	12.04	
Mexican American	25.33	10.52	
Other Hispanic	4.64	1.90	
Other ethnicity	3.79	3.32	
Education (%)			0.003
Lower than high school	29.37	24.36	
High school	23.03	24.74	
More than high school	47.59	50.90	
Physical activity (%)			< 0.001
Sedentary	21.85	28.63	
Low	28.22	26.54	
Moderate	18.54	18.48	
High	31.39	26.35	
Total protein (g/dL)	7.37 ± 0.48	7.20 ± 0.49	< 0.001
Total cholesterol (mg/dl)	200.50 ± 42.40	208.92 ± 41.51	< 0.001
HDL cholesterol (mg/dl)	51.93 ± 15.41	55.14 ± 17.07	< 0.001
Triglycerides (mg/dl)	141.95 ± 129.97	155.89 ± 109.26	< 0.001
Hemoglobin (g/dL)	14.46 ± 1.51	14.04 ± 1.39	< 0.001
Serum albumin (g/dL)	4.32 ± 0.33	4.21 ± 0.32	< 0.001
Calf circumference (cm)	38.18 ± 4.05	38.46 ± 4.26	0.058
Smoking status (%)			< 0.001
Non-smoker	53.10	46.35	
Smoker	46.90	53.65	

Mean ± SD for continuous variables and *P* value was calculated by Kruskal–Wallis test. % for Categorical variables and P value was calculated by weighted chi-square test.

*OA* osteoarthritis.

### Association between sarcopenia and OA

Table 3 showed the association between sarcopenia and OA. When we examined sarcopenia that was defined using BMI-adjusted SMI, sarcopenia was significantly associated with occurrence of OA in Model 1 (OR = 2.20 [95% CI, 1.89, 2.57]). These associations persisted after further adjusting for age, gender, and race/ethnicity in Model 2 (OR = 1.18 [95% CI, 0.98, 1.40]) and additionally adjusting for education, physical activity, total protein, total cholesterol, HDL cholesterol, triglycerides, hemoglobin, serum calcium, and calf circumference in Model 3 (OR = 1.23 [95% CI, 1.01, 1.51]). When we examined sarcopenia that was defined using weight-adjusted SMI, we also found that sarcopenia was significantly associated with OA after fully adjusting for covariates in Model 3 (OR = 1.30 [95% CI, 1.10, 1.55]).

**Table 3:** Association of sarcopenia with the risk of OA among participants in the NHANES 1999–2006 Cycles.

	OR (95% CI) *P* value		
	Model 1	Model 2	Model 3
Sarcopenia defined SMI by ASM/BMI			
OA (−) (n = 10,401)	1	1	1
OA (+) (n = 1055)	2.20 (1.89, 2.57) < 0.001	1.18 (0.98, 1.40) 0.074	1.23 (1.01, 1.51) 0.038
Sarcopenia defined SMI by ASM/Wt			
OA (−) (n = 10,401)	1	1	1
OA (+) (n = 1055)	3.18 (2.80, 3.62) < 0.001	1.54 (1.33, 1.79) < 0.001	1.30 (1.10, 1.55) 0.003

Model 1: no covariate was adjusted.

Model 2: age, gender, race/ethnicity were adjusted.

Model 3: additionally adjusted for education, physical activity, total protein, total cholesterol, HDL cholesterol, triglycerides, hemoglobin, serum calcium, calf circumference, and smoking status.

*CI* confidence interval, *OR* odds ratio.

**Figure 2:**
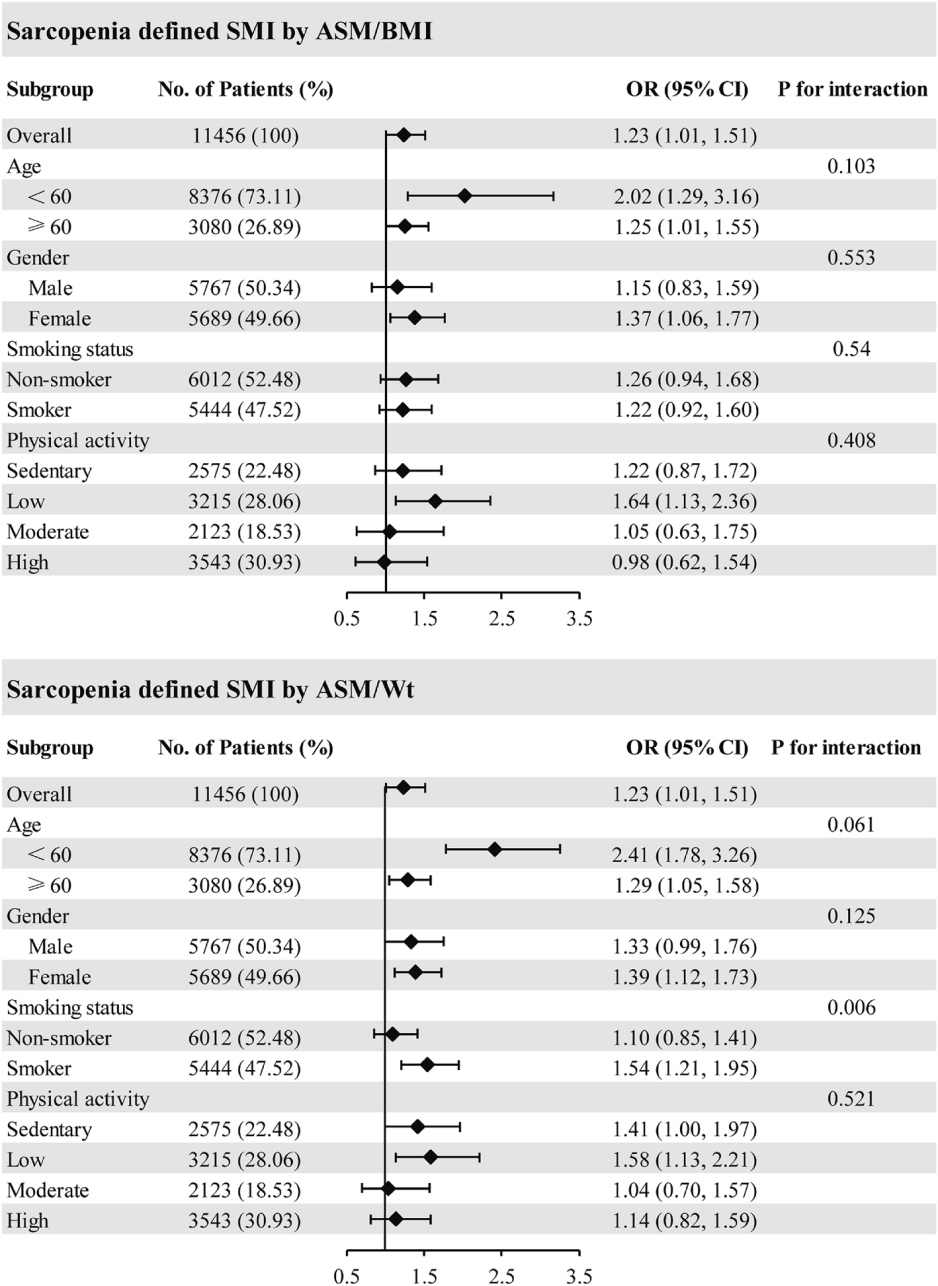
Association between Sarcopenia and osteoarthritis (OA) in different subgroups. Age, gender, and race/ethnicity, education, physical activity, total protein, total cholesterol, HDL cholesterol, triglycerides, hemoglobin, serum calcium, calf circumference, and smoking status were adjusted (the stratified variable was omitted from the model).

Subgroup analysis was conducted to examine whether the association between sarcopenia and OA were consistent among different population groups (Fig. 2). Sarcopenia (using the BMI-adjusted SMI) was associated with OA in populations of female (OR = 1.37 [95% CI, 1.06, 1.77]), and with low physical activity level (OR = 1.64 [95% CI, 1.13, 2.36]). When stratified by gender, these association was found among females (OR = 1.37 [95% CI, 1.06, 1.77]), not in males. Meanwhile, sarcopenia (using the weight-adjusted SMI) was associated with OA in populations of female (OR = 1.39 [95% CI, 1.21, 1.73]), smoker (OR = 1.54 [95% CI, 1.21, 1.95]), and with low physical activity level (OR = 1.58 [95% CI, 1.13, 2.21]). When we examined sarcopenia that was defined using weight-adjusted SMI, the interaction with the smoking status is remarkable (Pint = 0.006), indicating that the association between sarcopenia and risk of OA was more pronounced in smoker than non-smokers. The interactions with other group are not statistically significant.

## Discussion

In the present study, we used the nationally representative and large sample of US adults aged 20–85 years to investigate whether sarcopenia was associated with OA. We defined sarcopenia by using BMI-adjusted SMI and weight-adjusted SMI, and it was demonstrated that the association was consistent in both tow different definition. We found that sarcopenia was associated with occurrence of OA after fully adjusting for covariates. Subgroup analysis revealed that the association between sarcopenia and OA was more pronounced in smoker.

Sarcopenia is a degenerative disorder characterized by a progressive loss of muscle mass, strength, and function^16^. OA is a chronic joint disease characterized by joint degeneration and secondary hyperplasia, with joint pain, limited mobility, and joint deformity as the main symptoms^17^. Sarcopenia and OA usually occurs as an age-related process in older people. They share multiple risk factors, such as decreased estrogen levels, age, and obesity^18–20^. A cross-sectional study reported that obesity with sarcopenia had greater effect on knee OA compared to obesity without sarcopenia^21^. On the contrary, another study observed that no significant association between sarcopenic nonobesity with OA^22^. Accordingly, we investigated the association between sarcopenia and OA through a large sample of US adults. In our study, participants with sarcopenia had a higher occurrence of OA after fully adjusting for covariates, the results were consistent with those studies from Korean populations^23^. On the other hand, the results was inconsistent with few prior studies^11,22^, we speculated that the different races of subjects included in study design and lack of consistency in definition of sarcopenia partly explains discordant results. In addition, previous studies only commonly defined sarcopenia using an SM/BMI-derived cutoff point or an SM/Wt-derived cutoff point^24,25^. Here, we defined sarcopenia by both SM/BMI and SM/Wt when detecting its association with OA. We found that sarcopenia was associated with OA, and the association was consistent in both tow different definition.

Previous studies have investigated the pathogenesis between sarcopenia and OA owing to their similar biological mechanisms. The decline of muscle strength is the main feature of sarcopenia, and is also considered to be a primary risk factor leading to OA. Decreased muscle strength can reduce keen joint stability and accelerate articular cartilage degeneration. A recent study in rat found that knee muscle atrophy caused the subchondral bone abnormal change and cartilage degeneration, which revealed that decreased muscle strength would be a risk factor for development of osteoarthritis^26^. Inflammation may contribute to the pathogenesis of both sarcopenia and OA. The increase of pro-inflammatory cytokines leads to the imbalance of the protein synthesis and decomposition in muscles and cartilage, ultimately leads to muscle loss and cartilage destruction. Another study in rabbit model showed that Botulinum Toxin A induced joint instability in a muscle weakness model leads to elevations in a subset of pro-inflammatory cytokines in the synovial tissue and histological changes in cartilage, suggesting that sarcopenia may involve in the pathological process of OA by inducing inflammatory response^27^. In addition, irisin is involved in both sarcopenia and OA. Previous study observed that a low level of circulating irisin is a sensitive marker for muscle weakness and atrophy, and could help predict the onset of sarcopenia^28^. Meanwhile, irisin levels in serum and synovial fluid were negatively correlated with the severity of OA^29^.

In subgroup analysis and interaction analysis, we found that the association of sarcopenia (using the weight-adjusted SMI) and OA was more pronounced in smokers and the interaction with smoking status is statistically significant. It has reported that cigarette smoke extract mediates the disruption of cartilage through inducing cell death by increasing oxidative stress^30^. A recent Mendelian randomization study conducted by Ni et al.^31^ supported an independent deleterious causal effect for smoking upon OA risk, suggesting that strengthen smoking cessation interventions can lessen the burden of OA. Although we observed the interaction with smoking status is significant when sarcopenia defined by the weight-adjusted SMI, more prospective studies are warranted to validate the association among different smoking status group.

The biggest merit of this study is that the NHANES database contains a large and representative samples of the multi-ethnic population, which supports us to control for a large number of variables and to better conduct subgroup analyses. However, several limitations need to be considered when interpreting our findings. Firstly, it is difficult to explicit the causality between sarcopenia and OA due to the nature of the cross-sectional design. Secondly, the diagnosis of sarcopenia includes loss of muscle mass and muscle strength. However, data on muscle strength were not available in most participants and we only defined sarcopenia relied on low muscle mass which widely used in previous studies. Thirdly, the OA status, smoking status, and physical activity were assessed by questionnaire survey, making our data susceptible to recall and information biases. In addition, though we adjusted for several potential confounding variables associated with sarcopenia and OA, residual confounding is possible.

## Conclusion

In conclusion, our founding demonstrated that sarcopenia was associated with occurrence of OA. Subgroup analysis revealed that the association between sarcopenia and OA was more pronounced in smoker. Further well-designed prospective cohort studies are needed to assess our results.

## Data Availability

Publicly available datasets were analyzed in this study. This data can be found here: www.cdc.gov/nchs/nhanes.

## Abbreviations

NHANES: National Health and Nutrition Examination Survey
SMI: Skeletal muscle index
BMI: Body mass indexes
OA: Osteoarthritis
OR: Odds ratio
DXA: Dual-energy X-ray absorptiometry
ASM: Appendicular skeletal muscle
CI: Confidence interval
SD: Standard deviation
